# Loss of Sigma-1 Receptor Chaperone Promotes Astrocytosis and Enhances the Nrf2 Antioxidant Defense

**DOI:** 10.1155/2017/4582135

**Published:** 2017-08-14

**Authors:** Tzu-Yu Weng, Denise T. Hung, Tsung-Ping Su, Shang-Yi A. Tsai

**Affiliations:** ^1^Genomics Research Center, Academia Sinica, Taipei, Taiwan; ^2^Cellular Pathobiology Section, Integrative Neuroscience Research Branch, Intramural Research Program, National Institute on Drug Abuse, Department of Health and Human Services, National Institutes of Health, Baltimore, MD 21224, USA

## Abstract

Sigma-1 receptor (Sig-1R) functions as a chaperon that interacts with multiple proteins and lipids and is implicated in neurodegenerative and psychiatric diseases. Here, we used Sig-1R KO mice to examine brain expression profiles of astrocytes and ubiquitinated proteins, which are both hallmarks of central nervous system (CNS) pathologies. Our results showed that Sig-1R KO induces increased glial fibrillary acidic protein (GFAP) expression in primary neuron-glia cultures and in the whole brain of fetus mice with concomitantly increased accumulations of ubiquitinated proteins. Astrogliosis was also observed in the neuron-glia culture. Upon proteasome or autophagy inhibitor treatments, the pronounced ubiquitinated proteins were further increased in Sig-1R KO neurons, indicating that the Sig-1R regulates both protein degradation and quality control systems. We found that Nrf2 (nuclear factor erythroid 2-related factor 2), which functions to overcome the stress condition, was enhanced in the Sig-1R KO systems especially when cells were under stressful conditions. Mutation or deficiency of Sig-1Rs has been observed in neurodegenerative models. Our study identifies the critical roles of Sig-1R in CNS homeostasis and supports the idea that functional complementation pathways are triggered in the Sig-1R KO pathology.

## 1. Introduction

Sigma-1 receptor (Sig-1R) is a transmembrane chaperone protein that resides in the endoplasmic reticulum (ER). Enriched in the mitochondrion-associated ER membrane (MAM) domain where there are ample cholesterol and lipids [1], the Sig-1R has been implicated in many neurodegenerative and psychiatric diseases, as well as in drug-abuse-regulated synaptic plasticity [2–4]. As a chaperone protein, the Sig-1R interacts with ion channels, receptors [5], and lipids [6] and is noted for its interaction with another ubiquitously expressed chaperone, BiP (also known as GRP78), during the resting state. Upon stress induction or ligand stimulation, the Sig-1R disassociates from BiP and translocates to other cellular compartments to regulate ion channels or other receptor activities, such as inositol 1,4,5-trisphosphate type 3 (IP3) receptor activity [3, 7]. Sig-1Rs support cellular survival by maintaining mitochondrial homeostasis, Ca^2+^ concentrations [8], and ER stress responses [9]. The Sig-1R can translocate to the plasma membrane proximity upon ligand stimulation, modulates cyclin-dependent kinase 5 (cdk5) activity via lipid modifications [10], or translocates to the nuclear membrane to regulate gene transcription [11]. The Sig-1R plays promising roles not only in neuroprotection but also against other diseases [12]. Sig-1Rs are expressed higher in cancer tissues and correlated to decreased patient survival [13, 14] by influencing Ca^2^^+^ homeostasis and are participating in the apoptosis-induced caspase activation [15]. Sig-1R antagonists also show combined effects against cancers through ER stress and reactive oxygen species (ROS) as well as the induction of the apoptotic pathways [16]. In addition, Sig-1Rs function in lipid transport and regulations [6, 17], autophagy [18, 19], and inflammatory responses [3, 20].

The Sig-1R exists abundantly both in neurons and glia, implying that Sig-1Rs are involved in multiple physiological and pathological processes. Several lines of evidence support the hypothesis that the Sig-1R plays an important role in neuroprotection [21, 22]. Silencing of Sig-1Rs in primary neurons resulted in decreased mitochondrial membrane potential and aberrant formation of mitochondrial aggregates [10, 23], reduced spine formation, enhanced superoxide productions, hampered axonal extension [10, 23], reduced axonal density, and induced accumulation of phosphorylated tau and phosphorylated neurofilaments [10]. These phenomena are closely related to the stress conditions that characterize certain neurodegenerative diseases. Sig-1Rs have been linked to certain patients with neurological disorders. Namely, mutations of the Sig-1R were found in amyotrophic lateral sclerosis (ALS) cohorts [24, 25], and reduced density of Sig-1Rs was observed in the cerebral and cerebellar regions of the brain in early Alzheimer's disease (AD) patients [26]. In animal models, Sig-1R KO mice exhibit the phenotypes of motor neuron degeneration, reduced ER-mitochondrial contacts, and perturbation of mitochondrial and calcium homeostasis [27]; depletion of Sig-1R in the SOD1^∗^G93A or SOD1^∗^G85R mouse also accelerates ALS progression and is accompanied by MAM disruption [25, 28]. In addition, the Sig-1R agonist PRE-084 can alleviate the pathological and behavioral defects in experimental models of Parkinson's disease (PD) and ALS via upregulating neurotrophic factors or restoring Sig-1R's functions [25, 29]. Here, we sought to investigate hallmarks of neurodegeneration in the Sig-1R KO brain.

Astrocytes are functionally diverse in the central nervous system (CNS) and are gaining recognition in the pathological processes of neurodevelopmental and neurodegenerative diseases [30]. Emerging evidence indicates that astrocytes not only serve as supporting cells to maintain neuronal activity and survival, but are also involved in maintaining the integrity of the CNS. Upon brain insults, astrocytes are recruited to the injured sites where they may provide neuroprotective effects [31]. The double-edged sword role of astrocyte activation and overactivated gliosis accompanies many CNS pathologies [32]. In neurodegenerative diseases, reactive gliosis or astrocytosis [32] and enhanced glial fibrillary acidic protein (GFAP) expression are occurring in concert with neuronal degeneration [33]. Inhibition of the Sig-1R reduced the pathological activation of astrocytes in mechanical allodynia [34]. In the striatum, Sig-1Rs can activate astrocytes under methamphetamine treatment conditions via a self-activation mechanism [35], suggesting a complex yet important role of Sig-1R in balancing astrocyte activity in brain hemostasis. Sig-1Rs also regulate the oxidative stress system in the retinal glia cells via the Nrf2- (nuclear factor erythroid 2-related factor 2-) dependent system [36].

Production of excess ROS or imbalance of the antioxidative system is closely related to neurodegenerative disease [37]. A previous report found that the antioxidant protein Prdx6 was increased in the liver of Sig-1R KO animals compared to that of the WT mice [38]. Microarray data also revealed that silencing of Sig-1Rs in primary hippocampal neurons induced expression of genes related to the Nrf2-mediated oxidative stress pathway [39]. The Nrf2 is a transcription factor that binds to the antioxidant response element (ARE) and controls genes that participate in cellular defense against oxidative insults [40, 41]. Additionally, the Sig-1R has been shown to be involved in the functional activity of Nrf2 and ARE signaling pathways in retinal glia cells, liver, and lung tissues [36, 38]. We also examined the Nrf2 expression patterns in CNS-derived primary cultures from our Sig-1R KO system.

We previously reported that knocking down of Sig-1R increased the total amount of ubiquitinated proteins in neuronal cultures [10]. Sig-1R KO mice also display shorter axons and diminished axonal density compared to control mice [10]. To address the role of Sig-1Rs in neurodegeneration, we investigated the phenotype of Sig-1R KO mice. We hypothesize that the level of Sig-1Rs is critical in neurodegeneration by either invoking the activation of astrocytes or Nrf2. Characterization of the CNS or primary cultures from Sig-1R KO mice showed astrogliosis in addition to accumulation of ubiquitinated proteins, which may contribute to Sig-1R-deficiency-related pathology. We also observed an enhanced Nrf2 expression in Sig-1R KO HEK cells, neuronal-glial cultures, and astrocytes. This study supports the idea that functional complementation of opposing signaling pathways concerning cellular survival is triggered in Sig-1R KO mice.

## 2. Materials and Methods

### 2.1. Sig-1R Knockout Mice

Sig-1R KO mice were generated as previously described [10]. We obtained the mice of Oprs1 mutant (+/−) OprsGt(IRESBetageo)33Lex litters on a C57BL/6J×129s/SvEv mixed background from the Mutant Mouse Regional Resource Center of the University of California, Davis. The Sig-1R (+/−) males were backcrossed for 10 generations to females on C57BL/6J and then, mice were further genotyped. Mice were maintained in a 12-hour day/night cycle facility with free access to food and water. All animal studies adhered to protocols approved by the National Institutes of Health and the National Institute on Drug Abuse Intramural Research Program Animal Care and Use Program, which is accredited by the Association for Assessment and Accreditation of Laboratory Animal Care International.

### 2.2. Cells and Treatments

Primary cortical neurons were isolated as previously described [23]. Briefly, E18 fetus cortical neurons were dissected and incubated in 0.05% trypsin-EDTA for 15 min followed by trypsin inhibitor neutralization. After centrifugation, the supernatant was removed and the cell pellet was resuspended in a Neurobasal medium supplemented with B27, penicillin/streptomycin, and GlutaMAX-I (Thermo Scientific). A Pasteur pipette was used to dissociate the cells, and then, a cell strainer was used to separate the cells from tissue debris. Neuronal cultures were plated on a dish coated with poly-D-lysine hydrobromide and incubated in a 37°C, 5% CO_2_ incubator. B27 supplement without the antioxidants (AO) (Thermo Scientific) was used in this study to induce oxidative stress. Astrocytes were isolated according to the modified procedure [42]. Briefly, the isolated brain from 1-day-old neonatal mice was minced by a razor and incubated in 0.25% trypsin for 10 min with occasional shaking every 2 min. DNase was added prior to neutralization trypsin using fetal bovine serum (FBS). After centrifugation, the supernatant was removed and the cell pellet was resuspended in DMEM containing 10% FBS and penicillin/streptomycin followed by filtration with a cell strainer. Cells were plated on flasks coated with poly-D-lysine hydrobromide and incubated in a 37°C, 5% CO_2_ incubator. After 10 days, cells were vigorously shaken and washed to remove microglia cells. Remaining cells were trypsinized and transferred to new flasks. After 7 days, the flasks were vigorously shaken and washed, and the cells were trypsinized and transferred to plates coated with poly-D-lysine hydrobromide for further assays. CHO, Neuro-2a, or HEK cells were cultured in MEM or DMEM containing 10% FBS and penicillin/streptomycin and maintained at a 37°C, 5% CO_2_ incubator. PolyJet reagent (SignaGen Laboratories) was used for Sig-1R EYFP or Flag-Nrf2 [43] (Addgene) plasmid DNA transfections. For the reagent treatments, primary cultures or cell lines were treated with 10 *μ*M MG132 (Sigma), 1 *μ*g/ml cycloheximide (CHX) (Sigma), and 50 *μ*M chloroquine (CQ) (Sigma).

### 2.3. Western Blotting

Cells were washed with PBS and the lysate was collected on ice by a cell scraper with RIPA buffer (50 mM Tris, 150 mM NaCl, 0.2% sodium deoxycholate, 0.1% SDS, 1% Triton X-100, and adjusted to pH 7.2) supplemented with protease/phosphatase inhibitors and MG132; following collection, the lysates were sonicated and then centrifuged at 14000 rpm for 10 min at 4°C. Whole brain tissue was also lysed with the RIPA buffer containing protease/phosphatase inhibitors and MG132 followed by sonication. Brain tissue homogenates were collected after centrifugation at 14000 rpm for 10 min at 4°C. Protein concentrations were determined, and then, proteins were separated by SDS-PAGE and transferred to polyvinylidene difluoride membranes. Membranes were blocked with 5% nonfat milk for 1 h at room temperature and then incubated with the primary antibody overnight at 4°C. Membranes were then washed and incubated with the species-specific Alexa fluorescence secondary antibody (Thermo Scientific). Signals were acquired using the Kodak Image Station 4000 MM (Kodak) and analyzed by the Carestream MI or ImageJ software. Antibodies employed included the following: polyclonal anti-Sig-1R antibody produced by ourselves (1 : 1000), anti-GFAP antibody (number: ab2760, 1 : 5000) (Abcam), anti-ubiquitin antibody (number: 04-263, 1:1000) (Millipore), anti-Nrf2 antibody (number: 12721, 1 : 1000) (Cell signaling), anti-Flag antibody (number: F1804, 1 : 1000) (Sigma), anti-*β* actin antibody (number: A5441, 1 : 5000) (Sigma), anti-actin antibody (sc-1616, 1 : 1000) (Santa Cruz), anti-ERK antibody (number: sc-271270, 1 : 1000) (Santa Cruz), anti-tubulin antibody (number: T5168, 1 : 40000) (Sigma), and anti-Nup62 antibody (number: 610498, 1 : 2000) (BD).

### 2.4. Immunofluorescence

The cells were seeded on 12-well plates or coverslips coated with poly-D-lysine hydrobromide, fixed in 4% paraformaldehyde, and washed with 0.1% Triton X-100 in PBS. The fixed cells were blocked with 10% normal goat serum for 1 h at room temperature followed by incubation with primary antibodies at 4°C overnight. Cells were incubated with Alexa fluorescence secondary antibody (Thermo Scientific) for 1 h in the dark. Cells were then washed and kept in the PBS. Coverslips were further mounted with the antifading mounting reagent (Thermo Scientific). Images were taken using the Modular Laser System 2.0 (PerkinElmer) with the Nikon ECLIPSE TE2000-E confocal system (Nikon) or the Zeiss Axiovert 200 M microscope (Zeiss). Antibodies used in this research included the following: anti-GFAP antibody (number: ab2760, 1 : 5000; number: MAB360, 1 : 400) (Abcam; Millipore), anti-MAP2 antibody (number: MAB3418, 1 : 200) (Millipore), anti-LC3 antibody (number: 12741, 1 : 100) (Cell signaling), anti-p62 antibody (number: PM045, 1 : 500) (MBL), and anti-Nrf2 antibody (number: 12721, 1 : 100) (Cell signaling).

### 2.5. Immunoprecipitation

For the immunoprecipitation assay, cells were washed with ice-cold PBS and then crosslinked with 100 *μ*g/ml DSP in PBS on ice for 20 min. Tris-HCl (pH 8.8) was added at a final concentration of 50 mM to stop crosslinking for 15 min. Cells were washed and harvested in ice-cold PBS and then centrifuged (3500 rpm) for 15 min at 4°C. Cells were lysed in the RPIA buffer containing protease/phosphatase inhibitors and MG132, and the lysate was collected after centrifugation at 14000 rpm for 10 min at 4°C. The lysate was incubated overnight with the antibody of interest and then incubated with protein A/G PLUS beads (Santa Cruz) for 1.5 h. Beads were washed with modified RIPA buffer (50 mM Tris, 150 mM NaCl, 0.05% sodium deoxycholate, 0.05% SDS, 0.5% Triton X-100, and adjust to pH 7.2) containing protease/phosphatase inhibitors and MG132. Immunoprecipitated samples were preserved in the 2× Laemmli sample buffer (Bio-Rad) containing 2-mercaptoethanol. Antibodies employed included the following: anti-Nrf2 antibody (number: 12721, 1 : 50) (Cell signaling), anti-Flag antibody (number: F1804, 1 : 100) (Sigma), normal mouse IgG (number: sc-2025) (Santa Cruz), and normal rabbit IgG (number: sc-2027) (Santa Cruz).

### 2.6. Nuclear Fractionation Assay

WT and Sig-1R KO HEK cells (generated using the CRISPR KO system) were seeded onto the dish for 80% confluency overnight and then transfected with Flag-Nrf2 plasmid using PolyJet reagent. DMSO or 10 *μ*M MG132 was treated for 24 h after transfection. After 24 h of MG132 treatment (48 h posttransfection), nuclear fractions were collected with the Subcellular Protein Fractionation Kit for Cultured Cells (number: 78840, Thermo Scientific). Reagents were supplemented with the protease inhibitor and MG132, and the nuclear protein fractions were collected sequentially using the buffer provided according to the manual. Protein concentrations were determined and then subject to Western blotting.

## 3. Results

### 3.1. Mixed Neuronal-Glial Cultures Derived from Sig-1R KO Animals Are Prone to Astrocyte Activation

**Figure 1:**
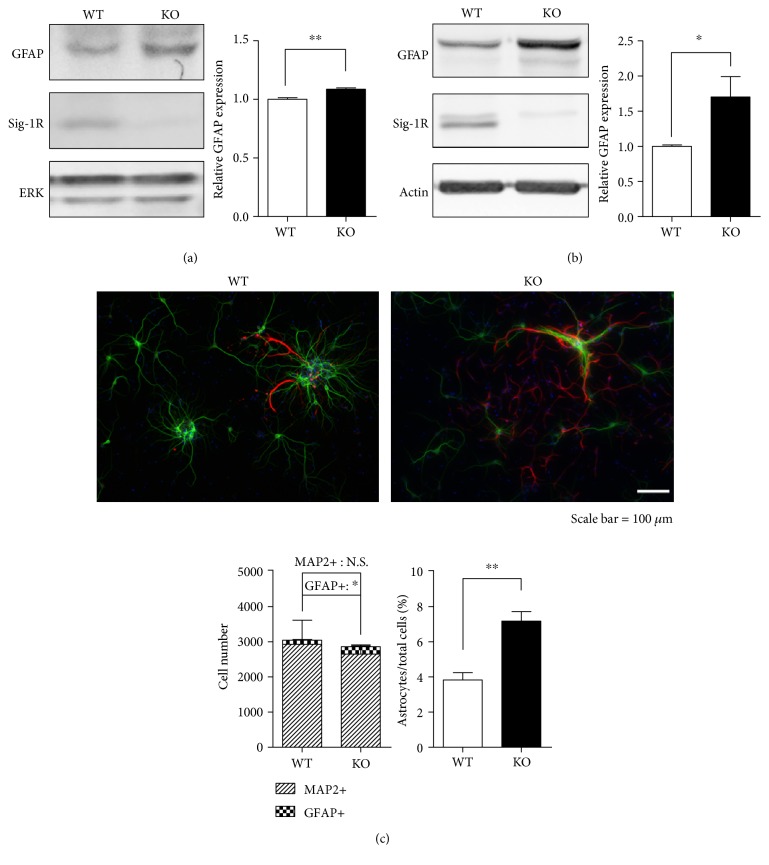
Pronounced astrocyte populations are observed in Sig-1R KO mixed neuronal-glial cultures. The whole brains of (a) E18 fetuses (*N* = 3) from Sig-1R WT or KO mice were collected and homogenized for GFAP immunoblotting. (b) Primary neuronal-glial cultures were isolated from Sig-1R WT or KO E18 fetuses. Cell lysates were harvested on DIV 11 for GFAP immunoblotting. Deficiency of the Sig-1R induced more GFAP expression in the fetus as well as in the primary neuronal-glial cultures. (c) Isolated mixed neuronal-glial cultures from Sig-1R WT or KO E18 fetuses were seeded at a density of 5 × 10^4^ cells per well and stained with the neuron and astrocyte markers MAP2 (green) and GFAP (red), respectively, on DIV 11. Sig-1R KO mixed neuronal-glial cultures have more GFAP-positive astrocytes. In the graph, GFAP was normalized to the internal control. The GFAP expression level in the WT was normalized to 1. Error bar indicates SEM. ^∗^*p* < 0.05, ^∗∗^*p* < 0.01, *t*-test.

To test whether gliosis occurs in Sig-1R KO mice, we examined expression of GFAP in the animal brains as well as in the primary mixed neuronal-glial cultures. Whole brain homogenates from E18 Sig-1R KO fetuses showed increased GFAP expression (Figure 1(a)). Enhanced GFAP expression was also observed in the Sig-1R KO mixed cultures compared to that of WT on DIV 11 (Figure 1(b)), suggesting a pronounced astrocyte population and increased astrocytic activities in the KO neuronal-glial cultures. Immunofluorescence data also revealed that Sig-1R KO neuronal-glial cultures exhibited increased numbers of GFAP-positive astrocytes and an increased GFAP ratio compared to the WT cells as both cultures were maintained under the same culture conditions since DIV 0 (Figure 1(c)). Primary cultures from KO animals also tended to express fewer MAP2-positive neurons. These data further support the concept that Sig-1R KO conditions favor astrocyte proliferation in mixed neuronal-glial cultures.

### 3.2. Sig-1R KO Brains Have Dysregulated Protein Degradation Systems

**Figure 2:**
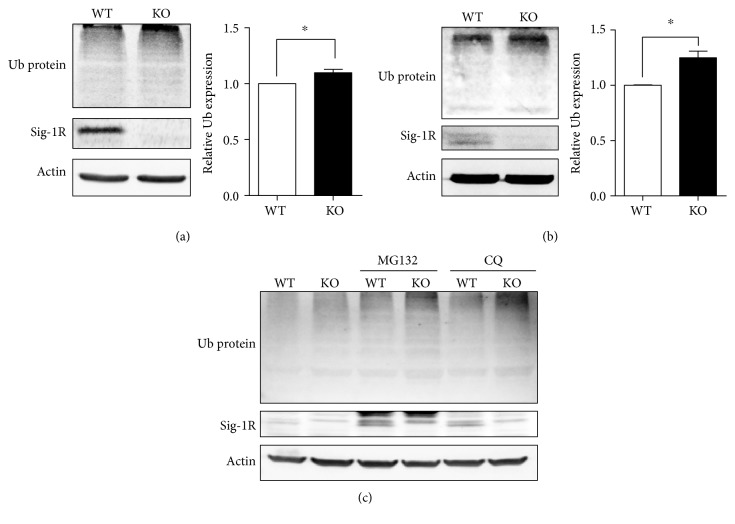
Sig-1R deficiency induced dysregulated protein degradation systems. The whole brains of (a) E18 fetuses (*N* = 3) or (b) 14 ~ 15-month-old adult mice (*N* = 3) from Sig-1R WT or KO mice were collected and homogenized for immunoblotting. Sig-1R KO homogenates showed more ubiquitinated protein expression. Briefly, Ub protein or Ub in this figure. In the graph, ubiquitinated protein was normalized to the internal control. For the ubiquitinated protein expression in the fetus brain, it was further normalized to the respective size-matched WT fetus as 1. The ubiquitinated protein expression level in the WT was normalized to 1. Error bar indicates SEM. ^∗^*p* < 0.05, *t*-test. (c) Mixed neuronal-glial cultures isolated from Sig-1R WT or KO E18 fetuses were treated with MG132 or CQ on DIV 11 for 24 h. Lysates were collected for immunoblotting, and ubiquitinated protein levels were increased in Sig-1R KO neurons, especially after the inhibitor treatments.

The pronounced astrocyte populations in Sig-1R KO mixed neuronal-glial cultures prompted us to examine ubiquitination levels in Sig-1R KO mice to tease out a potential mechanism that leads to astrocyte activation. Enhanced ubiquitination was observed in brains harvested from fetuses or adult mice (Figures 2(a) and 2(b)). To further define the pathological mechanism in Sig-1R KO neurons, mixed neuronal-glial cultures were treated with either the proteasome inhibitor MG132 or the autophagy flux inhibitor chloroquine (CQ). Pretreatment with MG132 or CQ further increased the levels of ubiquitinated proteins (Figure 2(c)) in Sig-1R KO primary cultures, suggesting that both protein degradation systems were dysregulated under Sig-1R-deficient conditions and implicating that the Sig-1R plays an important role in protein homeostasis. Sig-1R proteins also accumulated after the MG132 or CQ treatment. Proteins are either degraded through the classical ubiquitin-proteasome and the autophagy-lysosome pathways [44] although majority of proteins are degraded by the proteasome pathway. We next examined whether the Sig-1R is also degraded by the autophagy system.

### 3.3. Sig-1Rs and Autophagosomes Reside in Different Compartments

**Figure 3:**
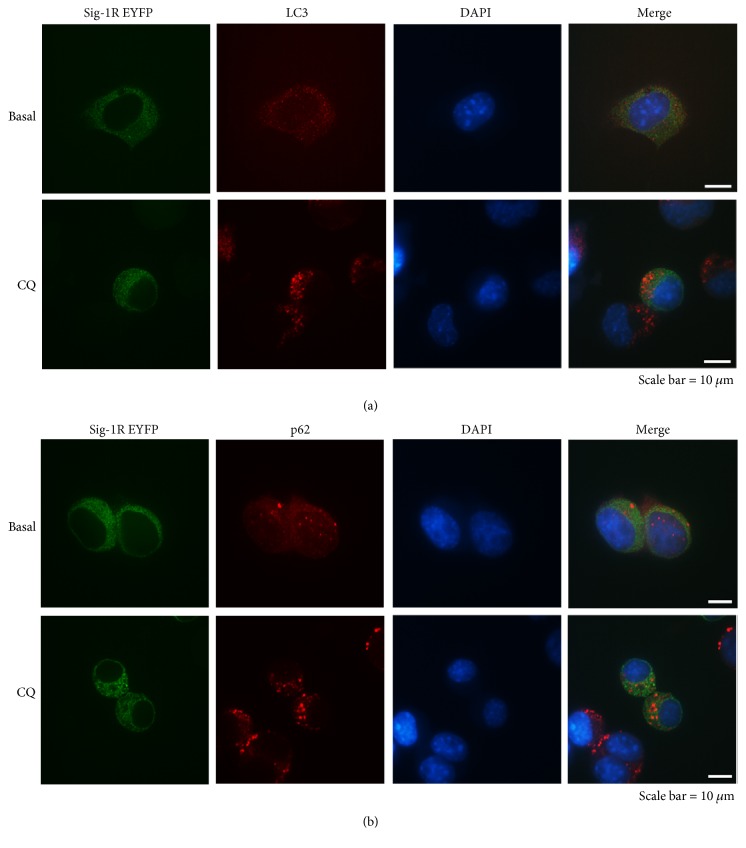
Sig-1R does not colocalize with autophagosomes. Neuro-2a cells were transiently transfected with Sig-1R EYFP for 24 h and followed by another 24 h of CQ treatments. Confocal images showed that the Sig-1Rs did not colocalize with (a) LC3, (b) p62, or the respective CQ-treated puncta.

To test whether the Sig-1R is degraded by autophagy, Neuro-2a cells overexpressed with EYFP-tagged Sig-1Rs were treated with CQ to distinguish the degradative stage of autophagy. The Sig-1R EYFP showed the classical diffused ER pattern as previously described [11] but did not colocalize with LC3 or p62 in untreated cells (Figure 3). LC3 is recruited to the inner and outer membranes of autophagosomes where inner-bound LC3 is degraded after subsequent lysosome fusion [45]. Additionally, p62 is degraded by autophagy [46]. We speculated that the Sig-1R is degraded by proteasomes but not by autophagosomes since Sig-1R EYFP does not colocalize with LC3 (Figure 3(a)) or p62 puncta (Figure 3(b)) in the CQ-treated cells. The Sig-1R displayed a slightly different distribution pattern in treated cells, where the Sig-1R resides in different compartments from the isolated autophagosomes. This raised the possibility that Sig-1Rs do not aid in protein degradation through the latter stages of autophagy.

Because we know that silencing Sig-1Rs enhances free radical production [23] and because Sig-1Rs have been shown to be involved in Nrf2-mediated oxidative signaling [36, 38, 39], we sought to examine the expression patterns of Nrf2 in Sig-1R KO neurons and how Nrf2 is regulated under oxidative stress conditions.

### 3.4. Nrf2 Signaling Is Enhanced in Sig-1R KO Neuronal-Glial Cultures upon Oxidative Stress

**Figure 4:**
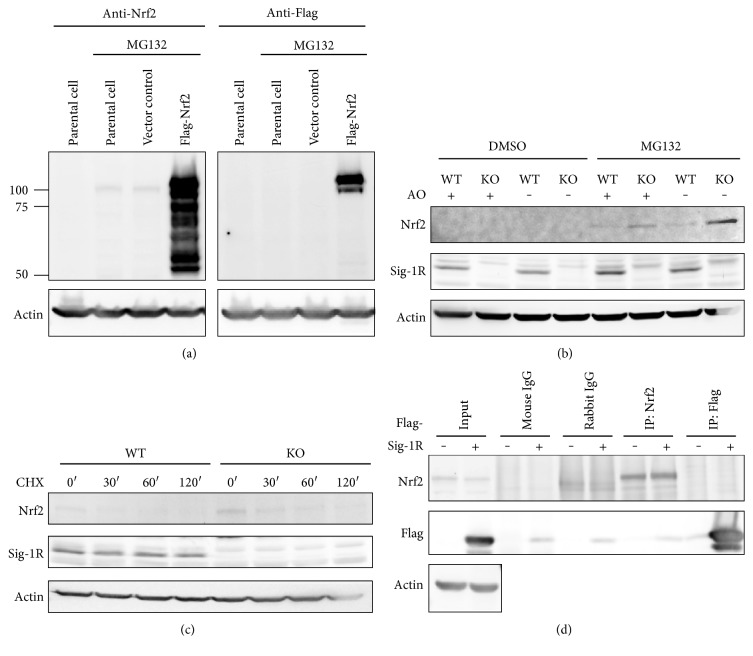
Sig-1R KO neuronal-glial cultures have enhanced Nrf2 expression. (a) CHO cells were transiently transfected with Flag-Nrf2 for 48 h and then treated with or without MG132 for 2 h. Cell lysates were harvested and immunoblotted with Nrf2 or Flag antibodies. Note that only cells pretreated with MG132 have detectable Nrf2 signals. (b) Mixed neuronal-glial cultures isolated from Sig-1R WT or KO E18 fetuses were treated with DMSO or MG132 on DIV 11 with or without AO supplements for 24 h. Cell lysates were collected, and the immunoblot showed that Nrf2 was increased in the KO primary cultures, especially when the Sig-1R KO cells were incubated in the AO-free medium. (c) Mixed neuronal-glial cultures isolated from Sig-1R WT or KO E18 fetuses were pretreated with MG132 for 2 h on DIV 11 followed by treatment with CHX. Cell lysates were collected at the indicated time points. (d) CHO cells were transiently transfected with Flag-Sig-1R for 48 h and then treated with MG132 for 2 h followed by immunoprecipitation. Nrf2 does not interact with Sig-1Rs.

The characterization of the Nrf2 proteins has been problematic in part due to the paucity of Nrf2 signals and in part due to the fact that the exact Nrf2 molecular weight observed by Western blotting analysis is still under debate [47]. We exogenously expressed Flag-tagged Nrf2 in CHO cells and analyzed the molecular pattern using Flag antibodies as well as the Nrf2 antibody. In our system, Nrf2 showed a molecular weight of approximately 100 kDa (Figure 4(a)). This result is in agreement with previous reports [47]. Notably, due to its quick turnover rate, Nrf2 was only detectable when cells were pretreated with proteasome inhibitors (Figures 4(a) and 4(b)); little or no Nrf2 was detectable in the control DMSO-treated cells. Therefore, cells were pretreated with MG132 to amplify Nrf2 expression levels throughout all experiments. To determine the expression of Nrf2 in Sig-1R KO neuronal-glial cultures, cells were pretreated with MG132 in combination with different conditioned media (culture medium supplemented with or without antioxidants (AO)). Our results showed that Sig-1R KO primary cultures exhibited higher Nrf2 levels than control cells. Additionally, Nrf2 levels were further enhanced in cells cultured without AO supplementation (Figure 4(b)). To analyze the Nrf2 protein stability, cycloheximide (CHX) was added to the primary cultures and cell lysates were collected at the indicated time points. There were faint Nrf2 signals in the WT group as observed in Figure 4(b), while a more pronounced signal was detected in the KO group, and it gradually decreased/diminished with 2 h of CHX treatment (Figure 4(c)). Since the half-life of Nrf2 is within 30 min, our data are consistent with previous reports that Nrf2 has fast degradation rates [48]. We also used an immunoprecipitation assay to examine the association between the Sig-1R and Nrf2. We found that these two proteins do not physically interact (Figure 4(d)), indicating that the Sig-1R does not regulate Nrf2 via a direct interaction. These results indicate that Nrf2 signaling is enhanced in Sig-1R KO neuronal-glial cultures by an as yet unknown mechanism.

### 3.5. Nrf2 Signaling Is Enhanced in Sig-1R KO Astrocyte and Cell Lines

**Figure 5:**
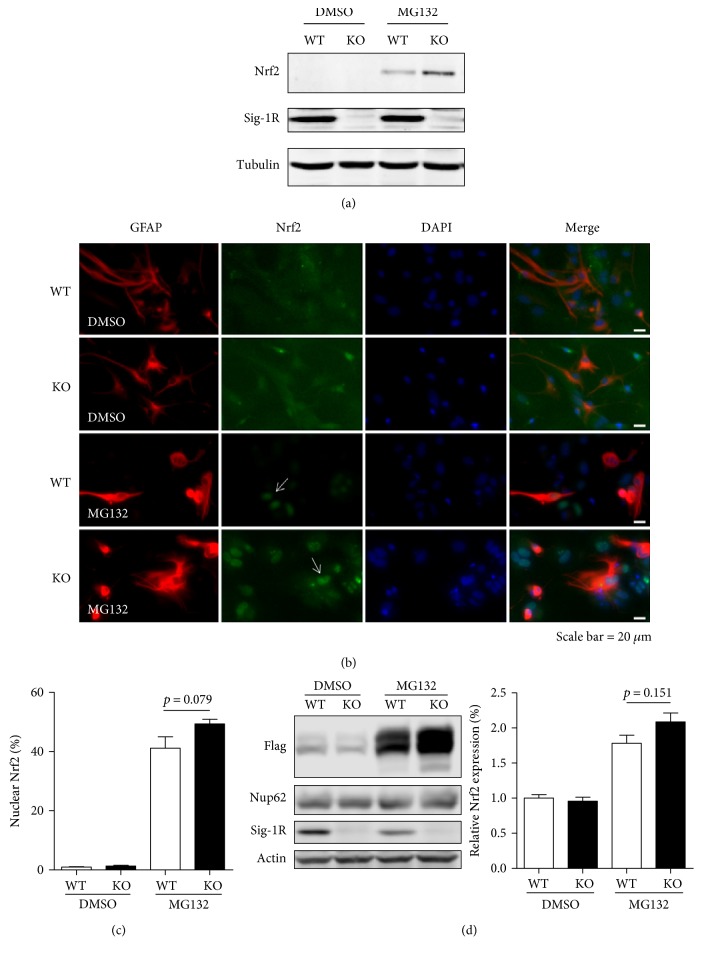
Nrf2 is enhanced in the Sig-1R KO cells under MG132 treatment. (a) Primary astrocytes isolated from Sig-1R WT or KO neonatal brains were treated with DMSO or MG132 for 24 h. Cell lysates were collected, and the immunoblot showed that Nrf2 was increased in the KO cells compared with WT cells under MG132 treatment. (b) Isolated astrocyte cultures from Sig-1R WT or KO neonatal brains were treated with DMSO or MG132 for 24 h followed by staining with the Nrf2 and GFAP antibodies. MG132 induced the pronounced nuclear pattern of Nrf2 in WT and KO cultures, as shown by the arrow. (c) To obtain nuclear Nrf2 percentage, the number of nuclear Nrf2 positive cells was divided by the total cell numbers (as indicated by DAPI signal). The images were captured from 3-4 random fields per sample. (d) WT or Sig-1R KO HEK cells were transiently transfected with Flag-Nrf2 for 24 h and then treated with DMSO or MG132 for another 24 h. Nuclear fractions were collected to determine the Nrf2 expression pattern. Nrf2 was normalized to the internal control. The Nrf2 expression level in the WT DMSO-treated group was normalized to 1. A trend of increased Nrf2 was observed in the Sig-1R KO cells. Statistical analysis was performed using *t*-test. Error bar indicates SEM.

Glia cells, especially astrocytes, have been demonstrated to preferentially protect neurons from the oxidative insults in the Nrf2-depedent pathway [49]. To further investigate the Nrf2 signaling in astrocyte under Sig-1R KO conditions, we isolated primary astrocytes from the 1-day-old neonatal mice. The Nrf2 expression level is enhanced in the Sig-1R KO astrocytes when compared with that in the WT astrocytes treated with MG132 (Figure 5(a)). Nrf2 is known to translocate into nucleus and activate ARE genes to combat the oxidative stress [40, 41]. We examined the Nrf2 expression pattern by immunofluorescence. Nrf2 was detected in the nucleus from WT or KO cells (Figure 5(b)) in the presence of MG132 while a weak nuclear Nrf2 signal was observed in the control DMSO-treated groups. Although the nuclear expression of the Nrf2 did not reach the statistical significance (*p* = 0.079) between the WT and Sig-1R KO astrocytes, there is a trend that the nuclear Nrf2 is increased in the Sig-1R KO astrocyte (Figure 5(c)), implying that astrocytes may need to activate Nrf2 in combating stress in Sig-1R KO condition. A different strategy was employed to further demonstrate that Nrf2 is increased in the nucleus. Exogenous Flag-Nrf2 was transfected to the WT or Sig-1R KO cells under MG132 treatments, followed by fractionation to separate nuclear protein. The results showed that Nrf2 exhibited abundantly in the nuclear fractionation and it dramatically accumulated after MG132 treatments in WT and KO cells where Nup62 was used as a nuclear marker (Figure 5(d)). Although the *p* value did not show statistical significance (*p* = 0.151), an increased trend of Nrf2 in the nuclear fraction was observed from Sig-1R KO cells compared to the WT cells under proteasome inhibition. The enhanced Nrf2 pattern is consistent with the ICC results. Those results suggest that the Nrf2 is upregulated and accumulated in the nucleus in mixed neuronal-glial cultures and astrocytes as well as in the cell lines when the Sig-1R is depleted from those cells under stress.

## 4. Discussion

**Figure 6:**
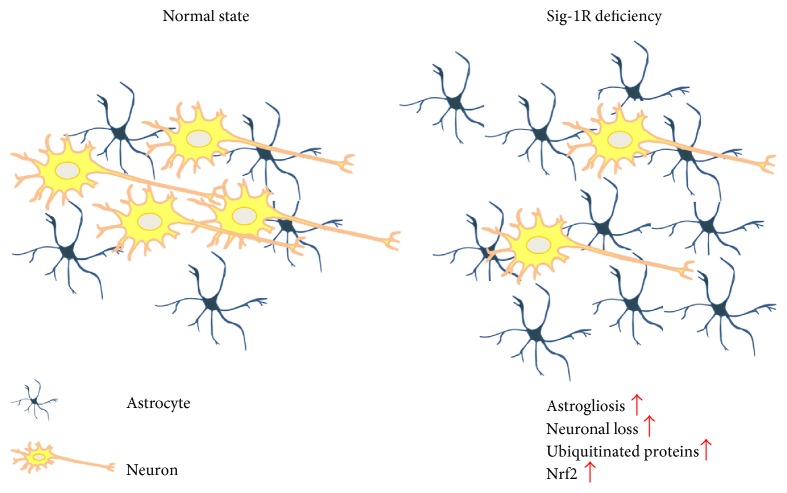
The proposed model for imbalanced interplay between neurons and astrocytes in Sig-1R deficiency pathologies. In the normal state, astrocytes maintain the homeostasis and functions of the neuronal cells. In the state of Sig-1R deficiency, an increased astrocyte population is seen at the early stage to cope with the stressful conditions, but this later becomes harmful to the neuronal cells and leads to neuronal loss. The ubiquitinated proteins are accumulated during this process and disrupt the proteostasis. The enhanced Nrf2 here may represent another compensatory mechanism via an as yet unknown mechanism.

It is widely accepted that Sig-1R-mediated cellular functions are associated with the pathogenesis of many neurodegenerative disorders. In this study, we found that Sig-1R KO animals are prone to have imbalanced astrocyte populations, dysregulated metabolism, and enhanced Nrf2 signaling. These impaired cellular events may be a consequence of compensatory mechanisms caused by a Sig-1R deficiency (Figure 6). Astrocytes play critical roles in neuronal homeostasis including modulating glutamate uptake and recycling, regulating ion and energy production, and exerting oxidative stress protection [50]. Sig-1R KO animals exhibit a lower neurofilament density [10]. In addition, depletion of Sig-1Rs creates an environment that is less favorable for neurons, causing neurons to become more vulnerable to cellular stress. Astrocytosis is observed in Sig-1R KO mixed neuronal-glial cultures (Figure 1), which could counterbalance a stressful microenvironment under the Sig-1R-deficient condition. Gliosis has been found to occur in the affected regions of the CNS as means from adjacent tissue to limit the lesion size and to stimulate neuroprotection in regulation of CNS homeostasis. However, if gliosis goes out of control, adverse effects on neuroplasticity may occur [32].

Thus, the astrocytosis that is propagated in Sig-1R KO neuronal cultures may serve to protect neurons or on the other hand to limit neuronal growth under unfavorable conditions. Astrocytes maintain neuronal homeostasis by providing neurons with energy and nutrients and by maintaining a suitable microenvironment for neurons for example by defending neurons against antioxidant insult via the Nrf2-dependent pathway [49, 51]. Another study has previously indicated that the Nrf2-induced gene upregulation signaling pathway was deficient in enriched neuronal cultures but was observed instead in astrocyte cultures, implicating the Nrf2 activation as well as the astrogliosis in Sig-1R-deficient cells [52]. Here, we demonstrated that Nrf2 is enhanced in both cell types, implying that neurons and astrocytes work cooperatively to survive under stressful conditions. Emerging evidence also shows that the Sig-1R mediates cellular functions via astrocyte activation. It has been demonstrated that Sig-1Rs can activate astrocyte functions through a positive-feedback mechanism under methamphetamine-stimulated conditions and the activation can be reversed by Sig-1R antagonist BD-1047 [35]. A recent study demonstrated that populations of activated GFAP-positive cells in Sig-1R KO mice were similar to those in WT mice in the substantia nigra. Interestingly, the study found that Sig-1R deficiency also led to reduction in MPTP-induced astrocyte activation [53]. Increased expression of GFAP in the cortex has been found in mice treated with cocaine for one week. Additionally, exposing astrocytes to cocaine resulted in rapid translocation of Sig-1Rs to the plasma membrane and activated downstream signaling pathways subsequently [54]. Chronic treatments of Sig-1R agonist PRE-084 decreased reactive astrocytosis in a motor neuron degeneration mouse model [21]. Another study also indicated that in Sig-1R KO impaired neurogenesis in the hippocampal dentate gyrus, suggesting that reduced survival without changes in the progenitor cell differentiation was seen in the absence of Sig-1R [55]. These studies indicate a critical role of Sig-1R in balancing astrocyte activation and neuron homeostasis. These become profound when the Sig-1R KO neurons are more vulnerable to cellular stress [10]. More experiments are warranted to tease out the brain regions that are more responsive to Sig-1R-mediated astrocytic regulation. Collectively, these data suggest that astrocytosis in Sig-1R KO brains is due to cellular responses that counterbalance neuronal damage via mechanisms yet to be discovered.

The increased accumulation of ubiquitinated proteins in Sig-1R KO neuronal-glial cultures becomes manifested when cells are treated with MG132 or CQ, suggesting that the metabolic regulation is compromised in Sig-1R deficiency when a stressful condition is imposed, such as ER stress or defective autophagy. The Sig-1R has been demonstrated as an ER sensor that facilitates interorganelle signaling to promote cell survival [9]; here, we also demonstrated that the Sig-1R plays an important role in proteostasis. An increase of Sig-1Rs was also observed after MG132 treatment (Figures 2(c) and 4(b)), we propose that Sig-1R may also be degraded through proteasome degradation system. Proteasome inhibition induced cell apoptosis, ROS production [56], and ER stress [57] and elevated the ER chaperones such as BiP [57, 58]. The Sig-1R can attenuate the ER stress [59]; therefore, we speculate that the cellular stress caused by the MG132 may lead to partial induction of Sig-1R expressions. Additionally, we observed that Sig-1Rs are accumulated after the CQ treatment (Figure 2(c)) but did not colocalize with autophagosomes (Figure 3()). We speculate that the upregulation of Sig-1Rs is induced due to the autophagy dysfunction. The accumulation of misfolded proteins affecting various cellsignaling systems is a shared hallmark in neurodegenerative diseases [60]. The Sig-1R participates in the degradation of proteins, such as UDP-galactose:ceramide galactosyltransferase [61], or intracellular inclusion, such as in Huntington's disease (HD) [62], via the ER-related degradation machinery. It is also interesting to note that the Sig-1R itself is accumulated and present in neuronal nuclear inclusions in various neurodegenerative diseases [63]. In vitro studies demonstrated that leptomycin B and thapsigargin-sequestered Sig-1Rs in the nucleus together with p62 [63]. We found that the Sig-1R was not colocalized with p62 under CQ treatment (Figure 3(b)). The Sig-1R has been reported to regulate autophagy [18, 19]. Sig-1Rs may interact with p62 differently in the proteasome and autophagy degradation systems. Studies have implied crosstalk between proteasome and autophagy degradation systems [44]. The protein folding and quality control system takes place in the ER [64], while autophagy organogenesis is suggested to originate at the MAM [65]. Given the specific location of Sig-1R in the ER at the MAM and facing the cytosol [66], it can be concluded that the Sig-1R is a multifunctional protein and plays important roles in both degradation systems.

Sig-1Rs exert neuroprotective actions to regulate free radical generation, oxidative stress, unfolded protein responses, immune responses, and survival pathways [67]. Earlier generations of homozygous Sig-1R KO mice had a lower survival rate after birth than the WT mice in our system. We previously demonstrated that silencing of Sig-1R results in accumulation of more ROS as compared to control CHO cells [68] or hippocampal neurons [23]. We believe that compensatory mechanisms, such as Nrf2 antioxidant signaling, might have been activated in the later breeds. The Sig-1R has been reported to modulate oxidative stress responses that are possibly involved in the regulation of neuroplasticity by Rac1 signaling [23], and treatment of bovine brain mitochondria with Sig-1R agonist- (+) pentazocine led to ROS production [69]. ROS scavengers prevented Sig-1R siRNA induced downregulation of Bcl-2, and silencing of Sig-1R also potentiated H_2_O_2_-induced cell death [70]. Reports also suggest that Sig-1R ligands such as PRE-084 can mitigate the effects of nitrosative and oxidative stress to proteins [71]. In a cellular model using Sig-1R KO Müller glia cells, Nrf2 expression and Nrf2-ARE-binding affinity were significantly decreased [36]. We speculate that these diverse results may be due to the employment of different cell types and experimental conditions. Transient silencing of Sig-1Rs in rat hippocampal neurons reduced the mRNA expression of HO-1 and other ARE-element response genes (data not shown). The results were consistent with studies supporting the idea that the Sig-1R regulates oxidative stress via Nrf2. Therefore, we speculate that the Sig-1R may contribute to the acute-phase response of Nrf2. A recent study also redefined the role of Nrf2, showing that Nrf2 inactivation was required for proper neuronal development, and ectopic expression of Nrf2 caused defective neuron morphology and synaptogenesis, which results from the imbalance of the redox system [72]. Furthermore, Sig-1Rs not only protect cell against cellular stress [38] but also regulate lipid metabolism or transportation while Nrf2 is also involved in lipid homeostasis [73]. Sig-1R ligands, PRE-084 and pentazocine, alleviate A*β* peptide-induced lipid peroxidation in hippocampus [74] and retinal lipid oxidation [75], respectively. Lipid peroxidation is also increased in Nrf2-compromised neurons [76]. However, the Sig-1R does not directly regulate or interact with Nrf2 (Figure 4(d)) in the resting condition, and the physical interaction between the multiple molecular partners [5] and Sig-1R may change when the cells are exposed to stressful circumstances. Our data indicated that other glia cells may also contribute to compensate the stressed condition as the nuclear Nrf2 pattern was also observed in GFAP-negative cells (Figure 5(b)). Furthermore, silencing of Sig-1R not only destabilized the lipid raft but also caused the defective autophagy [18], and dysregulation of autophagy has been shown to activate Nrf2 in a noncanonical pathway [77]. The enhanced Nrf2 signaling in Sig-1R KO neuronal-glial cultures, particularly astrocytes, may be a compensatory effect which promotes neuron survival and mitigates oxidative stress conditions.

It is possible that certain brain areas are more vulnerable to Sig-1R-deficiency-induced astrocyte activity. There may also be region-specific variations in astrocytic functions in response to stimulations. Sig-1R ligands have neuroprotective effects in ALS, AD, HD, PD, retinal degeneration, stroke, and methamphetamine-induced toxicity. These ligands may elicit neuroprotective effects via modulating ER homeostasis, balancing ROS, and both neuron and glial functions [78]. We demonstrated that Nrf2 is increased in the cell lines, the mixed neuronal-glia and astrocyte cultures from KO mice under stressed conditions. Our results suggest a potential compensatory mechanism caused by Sig-1R deficiency, addressing the important role of the Sig-1R in protein quality control systems and in the balance between neurons and glial cells.

## Abbreviations

AD: Alzheimer's disease
ALS: Amyotrophic lateral sclerosis
AO: Antioxidants
ARE: Antioxidant response element
Cdk5: Cyclin-dependent kinase 5
CHX: Cycloheximide
CNS: Central nervous system
CQ: Chloroquine
ER: Endoplasmic reticulum
GFAP: Glial fibrillary acidic protein
HD: Huntington's disease
IP3: 1,4,5-Trisphosphate type 3
MAM: Mitochondrion-associated ER membrane
Nrf2: Nuclear factor erythroid 2-related factor 2
PD: Parkinson's disease
ROS: Reactive oxygen species
Sig-1R: Sigma-1 receptor.
